# Epigenetics in Stroke Recovery

**DOI:** 10.3390/genes8030089

**Published:** 2017-02-27

**Authors:** Haifa Kassis, Amjad Shehadah, Michael Chopp, Zheng Gang Zhang

**Affiliations:** 1 Department of Neurology, Henry Ford Health System, Detroit, MI 48202, USA; haifa.kassis@gmail.com (H.K.); amjad.shehadah@gmail.com (A.S.); michael.chopp@gmail.com (M.C.); 2 Department of Physics, Oakland University, Rochester, MI 48309, USA

**Keywords:** epigenetics, stroke, recovery

## Abstract

**Abstract:** While the death rate from stroke has continually decreased due to interventions in the hyperacute stage of the disease, long-term disability and institutionalization have become common sequelae in the aftermath of stroke. Therefore, identification of new molecular pathways that could be targeted to improve neurological recovery among survivors of stroke is crucial. Epigenetic mechanisms such as post-translational modifications of histone proteins and microRNAs have recently emerged as key regulators of the enhanced plasticity observed during repair processes after stroke. In this review, we highlight the recent advancements in the evolving field of epigenetics in stroke recovery.

## 1. Introduction

Stroke remains a major health care challenge despite the increasing availability of acute thrombolytic interventions such as tissue plasminogen activator (tPA) and endovascular treatment strategies [1]. While the death rate from stroke has continually decreased due to interventions in the hyperacute stage of the disease, long-term disability and institutionalization have become common sequelae in the aftermath of stroke. Stroke costs the US economy $34 billion annually, including the costs of health care services, medications, and lost productivity [2]. Therefore, identification of new molecular pathways that could be targeted to improve neurological recovery among survivors of stroke is crucial. 

After stroke, restorative processes, such as angiogenesis, neurogenesis, oligodendrogenesis, synaptogenesis, and axonal outgrowth are induced in the areas adjacent to the infarct border and contribute to neurological recovery after ischemia [3,4]. The underlying mechanisms of these processes involve altered orchestrated expression of genes. Epigenetic mechanisms such as DNA methylation, post-translational modifications of histone proteins and microRNAs (miRNAs) regulate gene expression without directly changing the sequence of DNA. Considering the far-reaching effects of epigenetic mechanisms on gene regulation, they can potentially regulate key aspects of the enhanced plasticity observed during repair processes after stroke. In this review, we will highlight recent studies that have expanded our understanding of the roles of epigenetics in regulating stroke recovery. These exciting discoveries take us a step forward towards the development of epigenetic-based novel therapies aiming to improve neurological function among stroke patients.

## 2. DNA Methylation

Methylation of cytosine in CpG (cytosine-guanine) dinucleotides is a repressive epigenetic mechanism catalyzed by a family of DNA methyltransferase enzymes (DNMTs). DNMT1 is the most abundant DNMT in mammalian cells and binds to hemi-methylated DNA to maintain methylation, while DNMT3a and 3b bind unmethylated DNA and are responsible for de novo methylation [5]. 

DNMTs are expressed in postmitotic neurons [6] and the global level of DNA methylation increases in vivo acutely after mild ischemic injury/reperfusion. Administration of the broad-spectrum DNMT inhibitors 5-aza-2′-deoxycytidine and Zebularine to wild type rodents provides resistance and reduces ischemic injury which may indicate that DNA methylation contributes to cell death through silencing of neuroprotective genes [7,8,9]. Clinical studies have also shown that aberrant blood DNA methylation levels are associated with increased risk for atherosclerosis and stroke [10,11]. 

Studies have shown that DNA methylation is implicated in physiologic repair mechanisms such as adult neurogenesis and synaptic plasticity. For example, members of the methyl-CpG-binding proteins (MBDs) family of proteins which bind to methylated DNA play a role in the regulation of adult hippocampal neurogenesis [12]. MBD1 binds to methylation sites in the promoter of basic fibroblast growth factor 2 (FGF2) gene and loss of MBD1 impairs neural progenitor cells differentiation [13]. Methyl-CpG-binding protein 2 (MeCP2) is another MBD protein important in the formation of dendritic spines and synaptogenesis of hippocampal neurons [14]. Both MBD1 and MeCP2 are highly expressed in the brain and upregulated in the hippocampus at 24 h after stroke [15] which suggests a role for DNA methylation in the regulation of stroke-induced neurogenesis and synaptogenesis. 

## 3. Histone Acetylation in Stroke Recovery

The nucleosome is the basic unit of chromatin comprised of approximately 200 bp of DNA wrapped around an octamer of histone proteins [16]. Each histone octamer consists of two copies of each of the four core histone proteins (H2A, H2B, H3, and H4). Acetylation of lysine residues in histone proteins removes their positive charge resulting in detachment of the negatively charged DNA from histone proteins. Subsequently, acetylation leads to relaxation of chromatin (i.e., euochromatin), which is usually associated with increased gene transcription due to increased access to DNA promoter regions by transcription factors and RNA transcription machinery. Conversely, deacetylation restores the positive charge to histone proteins leading to compaction of chromatin (i.e., heterochromatin) and repression of gene expression [17]. 

Typically, histone acetylation and deacetylation states are controlled by maintenance of an appropriate balance between the enzymatic activities of histone acetyltransferases (HATs) and histone deacetylases (HDACs) [18]. Stroke induces a global reduction in acetylation levels of histones H3 [19,20,21] and H4 [22] in the ischemic brain. Histone hypoacetylation starts within hours and lasts at least up to two weeks [23] after the ischemic event. This disruption in the global acetylation homeostasis appears to occur due to a combination of increased activity of HDACs [24] along with decreased activity of HATs [25]. 

The observed histone hypoacetylation in the ischemic brain led to the hypothesis that HDAC inhibitors that increase histone acetylation levels may be a viable approach to treat stroke. Indeed, many non-selective HDAC inhibitors such as valproic acid (VPA) [20], suberoylanilide hydroxamic acid (SAHA) [26], 4-phenylbutyrate [27], sodium butyrate, and trichostatin A [19] have been tested in animal models of stroke and were shown to induce neuroprotection and reduce inflammation when administered within a few hours after stroke onset. Most recently, HDAC inhibitors have also been increasingly investigated as neurorestorative agents for stroke. Similar to their positive effects on hyperacute neuronal protection from cell death, delayed administration (24 h after the ischemic event) of non-selective HDAC inhibitors such as VPA and SAHA were found to improve functional outcome through increased axonal and dendritic outgrowth and white matter repair [24,28]. VPA treatment also enhances stroke-induced angiogenesis in animal models of ischemic stroke likely through upregulation of hypoxia-inducible factor 1-α (HIF-1α) and vascular endothelial growth factor (VEGF) [22]. Furthermore, non-selective HDAC inhibition with sodium butyrate induces brain-derived neurotrophic factor (BDNF)-receptor tropomyosin receptor kinase B (TrkB)-dependent stimulation of neurogenesis in the ischemic brain [23]. 

HDACs comprise a super-family of enzymes grouped into four major classes (Classes I–IV) based on their structure and homology to yeast enzymes [29]. The expression patterns of the different HDAC isoforms are differentially regulated by ischemia and show cell- and region-specific patterns seven days after stroke [22,30]. For example, oligodendrocyte progenitor cells in the peri-infarct white matter exhibit increased expression of HDAC1 and HDAC2, concurrent with increased proliferation, while mature oligodendrocytes, on the other hand, show decreased HDAC1 and increased HDAC2 [22]. These data suggest that individual HDAC isoforms within the same class may have differential effects on neurorestoration and oligodendrogenesis during brain repair after stroke.

The actions of HDACs are even more complex as some of the isoforms possess the ability to translocate between the cytoplasm and nucleus. For example, Class IIa HDACs (4 and 5) are mainly expressed in the cytoplasm under physiological conditions, but are also able to shuttle into the nucleus. In the nucleus, HDACs can access histone proteins and act as epigenetic regulators [31]. The role of nuclear HDAC4 in neuronal cell death and survival is controversial and highly debated in the literature. Previous studies have yielded contradictory results with some concluding that HDAC4 promotes cell death [32,33], while others have provided evidence that nuclear HDAC4 shuttling protects neurons from injury [34]. The controversy may stem from the diverse actions that HDAC4 may play depending on environmental cues under different in vitro conditions [35]. During recovery in vivo, stroke induces nuclear shuttling of HDAC4 (but not HDAC5) in neurons localized to the peri-infarct, and increased nuclear HDAC4 is strongly associated with endogenous neuronal repair, suggesting a positive role for HDAC4 in promoting neuronal recovery after ischemic injury [36]. HDAC remains highly localized to the nucleus up to at least four weeks after the ischemic event. Interestingly, unlike the non-selective HDAC inhibitors mentioned above, when the selective Class IIa HDAC inhibitor MC1568 was tested in vivo, it was found to increase mortality and lesion volume and impair neuronal remodeling after stroke; possibly through downregulation of phosphorylated cAMP response element-binding protein (CREB) and c-*fos* in neurons [24]. These studies further confirm the existence of isoform-specific roles for HDACs in endogenous brain recovery after ischemic stroke. Some isoforms such as HDAC4 exhibit restorative effects, while others such as HDAC6 [37] may promote cell death. Development of more selective inhibitor/activators of HDAC isoforms may provide a novel therapeutic approach that can potentially elucidate HDAC isoform specific functions and thereby facilitate enhanced brain recovery after stroke. 

Since very few studies in humans explored the roles of histone acetylation in stroke, most of our knowledge comes from experimental animal studies. However, in an effort to identify genetic factors that contribute to increased risk of stroke, a large genome-wide association study has identified that a variant in HDAC9 on chromosome 7p21.1 is associated with a 1.4-fold increase in risk for large vessel disease ischemic stroke [38]. HDAC9 expression was also found to be upregulated in human carotid plaques compared with controls [39]. Whether HDAC9 plays a role during recovery after stroke is currently unknown. 

Utilizing imaging technology to measure HDAC expression acutely and during recovery in stroke patients is clearly of great interest. A recent study shows that the expression of histone deacetylases in the human brain can be non-invasively imaged using Positron Emission Tomography (PET) imaging with [11C] Martinostat [40]. The authors found that HDACs are highly expressed throughout the healthy human brain and display region-specific distribution. This technology can potentially provide an additional tool for more clinical research in the area of histone acetylation.

Finally, many HDAC inhibitors are currently under intense investigation in clinical trials for their potential use as anticancer drugs [41]. The U.S. Food and Drug Administration (FDA) has approved a number of HDAC inhibitors including SAHA [42], romidepsin [43], belinostat [44], and panobinostat [45] for the treatment of cutaneous/peripheral T-cell lymphoma and multiple myeloma. Whether HDAC-based therapy will yield similar positive outcomes in stroke patients remains an open question that needs to be addressed in future clinical trials.

## 4. MicroRNAs in Stroke Recovery

MicroRNAs are abundant small (20–25 nucleotides) non-coding RNAs that regulate gene transcription via blockage of translation of messenger RNA (mRNAs) into proteins [46]. miRNAs are produced by a multi-step canonical mechanism that include transcription of a long hairpin-containing primary miRNA (pri-mRNA) by RNA polymerase II. The pri-miRNA is then cleaved by Drosha into pre-miRNA and exported into the cytoplasm by Exportin 5. In the cytoplasm, pre-miRNA is cleaved by Dicer and then binds with Argonaute (Ago) proteins in the RNA-induced silencing complexes (RISCs), which silence specific mRNA transcripts based on complementary to unique 3′ UTR sequence motifs [47]. 

miRNAs are highly expressed in the nervous system where they play key roles in development, physiology, and disease [48]. Considering the diversity of miRNA functions and their influence on a large number of neuronal and non-neuronal genes in experience and activity-dependent manners [49], it is not surprising that miRNAs have started to emerge as important players in stroke-induced endogenous brain recovery events such as angiogenesis, neurogenesis, oligodendrogenesis, and axonal outgrowth (Table 1).

The adult brain vasculature is activated in response to various pathological conditions including stroke [59]. Neurorestorative treatments, either cell-based or pharmacological therapies, in animal models of stroke induce angiogenesis, the formation of new blood vessels, which is associated with and may underlie improvements in neurological outcome [3]. Recent studies have indicated that specific miRNAs play key roles in endothelial function and angiogenesis [60]. Altered miRNA expression profiles were found in rat cerebral endothelial cells at seven days after stroke; 7 miRNAs (miR-225, miR-335, miR-139-5p, miR-203, miR-708, miR-193*, and miR-494) are downregulated, and another 9 miRNAs (miR-224, miR-210, miR-204, miR-322*, miR-100, miR-450a, miR-322, miR-331, and miR-101a) are upregulated. Of those, miR-139 and miR-335 are most strikingly downregulated at 60% and 90%, respectively; when the expressions of miR-139 and miR-335 are exogenously restored in cultured cerebral endothelial cells, they promote angiogenesis as measured by the capillary tube formation assay in vitro [50]. Similarly, another study has found that miR-15 is upregulated in endothelial cells up to 16 h after oxygen deprivation exposure in vitro and provided evidence suggesting that pharmacological inhibition of miR-15a may be a potential approach to increasing angiogenesis after stroke through targeting of basic fibroblast growth factor (bFGF) and VEGF in endothelial cells [51,52]. miR-155 is another key regulator of endothelial morphogenesis that was recently implicated as a potential therapeutic target for stroke. When a specific miR-155 inhibitor was administered two days after ischemia in an experimental mouse stroke model, it improved functional outcome, decreased vascular leakage, and promoted revascularization through stabilization of tight junctions and preservation of blood–brain barrier [53]. Another miRNA, miR-107, was also shown to be upregulated in the ischemic boundary zone on day 3 and day 7 after permanent middle cerebral artery occlusion in the rat and to contribute to post-stroke angiogenesis by targeting Dicer-1, an enzyme which regulates processing of miRNA precursors and regulates of VEGF in endothelial cells [54]. Taken together, these studies highlight the important roles of endothelial miRNAs and their potential as therapeutic targets for enhancing vascular remodeling and recovery after ischemia. 

In addition to endothelial cell activation in response to injury, the adult brain neural stem cells are also stimulated after ischemia. Stroke increases the proliferation rate of neural stem cells in the sub-ventricular zone (SVZ) of the lateral ventricle and induces migration of newly generated neuroblasts to the ischemic boundary area where they play a major role in promoting recovery after stroke [61,62]. miRNA profiling studies have revealed that miRNAs involved in the regulation of key signaling pathways in stem cell proliferation and differentiation such as Notch, Wnt, Sonic Hedgehog (*SHH*), and TGF-β are significantly altered after ischemia [63,64]. For example, miR-124a, the most abundant miRNA in neurons, is downregulated seven days after stroke and contributes to the dramatic increase in neural progenitor cell proliferation after ischemia [55]. The pro-proliferative effects of knocking down miR-124 are mediated through dampening of its inhibition of Jagged-1; Notch receptor ligand 1, a major promoter of neural stem cell proliferation after stroke [65]. Indeed, exogenously increasing the expression of miR-124 negatively impacts the proliferation of rat neural stem cells, suggesting that stroke-induced downregulation of miR-124 is an important mediator of neurogenesis after stroke.

miR-17, miR-18a, miR-19a, miR-20a, miR-19b-1, and miR-92a-1 are members of the miR-17-92 cluster, also known as oncomiR-1 due to its involvement in tumorigenesis [66]. In addition, this cluster also plays important roles in regulating neural progenitor cell proliferation and oligodendrogenesis during development [67,68]. Stroke-induced activation of the *SHH* pathway upregulates the expression of the miR-17-92 cluster in SVZ neural progenitor cells via c-Myc, one of the most potent oncogenic genes [56]. Overexpression of the miR 17-92 cluster members promotes stroke-induced neural progenitor cell proliferation possibly through suppression of phosphatase and tensin homolog *(PTEN)* deleted on chromosome 10, a tumor suppressor gene that negatively regulates cell proliferation [56]. Collectively, these studies highlight the roles of SVZ miRNAs in regulating stroke-induced neurogenesis. 

In addition to generation of new neuroblasts in the adult brain, SVZ progenitor cells also generate oligodendrocyte progenitor cells (OPCs) able to differentiate into mature oligodendrocytes after stroke [69]. miRs have been shown to play a pivotal role in regulating OPC proliferation and differentiation under physiologic conditions [70]. Recent studies have implicated miRNAs in regulating stroke-induced oligodendrogenesis. For example, miR-9 and miR-200b are downregulated in ischemic white matter at two weeks after ischemic injury and regulate stroke-induced oligodendrogenesis by targeting the transcription factor serum response factor (SRF) [57]. Another miRNA, miR-146a, is upregulated by stroke in the corpus callosum and SVZ of the ischemic hemisphere. Overexpression of miR-146a in neural progenitor cells in vitro significantly increased their differentiation into O4^+^ OPCs via inversely regulating its target gene inteleukin-1 receptor-associated kinase 1 (IRAK1). Furthermore, overexpression of miR-146a in primary OPCs in culture increases their expression of myelin proteins, whereas downregulation of endogenous miR-146a suppresses the generation of new myelin proteins [58]. Together, these data suggest that a number of specific miRNAs may mediate stroke-induced oligodendrogenesis. 

Recently, there is a growing interest in clinical studies in looking at whether circulating miRNAs can be used as diagnostic biomarkers in stroke patients. Several studies have demonstrated alterations in whole blood and plasma miRNA levels in stroke patients (extensively reviewed by Vijayan and Reddy [71]). Large numbers of miRNAs were found to be up or downregulated in acute stroke cases [72,73,74]. Of particular interest are levels of brain-specific miRNAs such as miR-124 and miR-9 [75] that could potentially aid clinicians in diagnosing stroke especially when magnetic resonance imaging is not available. However, the physiologic significance of miRNA blood levels relevant to injury and recovery mechanisms in the brain tissue needs to be investigated. Furthermore, miRNA-target interactions are cell type dependent [76]. The majority of studies have mainly analyzed miRNAs and their mRNA targets in bulk tissue samples of the brain [77,78]. The in vivo identification and functional analysis of miRNAs and their target miRNAs in neurons and glia are challenging after stroke.

## 5. Histone Deacetylases and microRNAs

miRNAs and HDACs may regulate each other in a manner that could critically coordinate their functions [79]. This relationship is not yet fully understood but could represent a pivotal interaction that influences gene expression and regulates restorative mechanisms during brain repair after stroke. One study has found that HDAC inhibition with VPA can alter the expression of a large number miRNAs in the ischemic cortex [80]. Interestingly, the predicted targets of these miRNAs include large networks of genes involved in the development of the vascular and nervous systems.

Other studies have found that miRNAs can regulate the expression of specific HDACs, and likewise, HDACs can also regulate the expression of specific miRNAs. For example, HDAC4 is negatively regulated by miR-206 in a model of amyotrophic lateral sclerosis [81], and in a Huntington’s disease model, miR-22 regulates HDAC4 [82]. On the other hand, SIRT1, a Class III HDAC, can regulate miR-134, and a loss of SIRT1 leads to increased miR-134 resulting in impairment in synaptic plasticity by a decrease in BDNF and CREB [83]. 

The interaction between HDACs and miRNAs in the ischemic brain during recovery has not been investigated, and studies to look into its role in brain repair processes are clearly warranted.

## 6. Conclusions

In this review, we highlight the recent knowledge in the evolving field of epigenetics in stroke recovery. Epigenetic players such as DNA methylation, HDACs, and miRNAs are potent modulators of gene regulation, and an accumulating body of evidence suggests that they play a pivotal role in regulating brain remodeling after stroke. While further studies to elucidate the functions of specific HDACs, miRNAs, and the interplay between them are warranted, epigenetic-based therapies carry a tremendous potential to serve as a novel multifaceted approach to enhance recovery among stroke survivors.

## Figures and Tables

**Table 1:** Key miRNAs altered by stroke and their potential roles in ischemic brain repair processes.

Brain Repair	miRNA	Effects in Ischemia	References
Angiogenesis	miR-139; miR-335	Downregulated after stroke; promote angiogenesis in vitro when upregulated	[50]
	miR-15a	Upregulated after stroke; promotes angiogenesis when blocked via targeting of BDNF and VEGF	[51,52]
	miR-155	In vivo inhibition of miR-155 leads to revascularization and BBB preservation	[53]
	miR-107	Upregulated in ischemic boundary zone in vivo; contributes to post-stroke angiogenesis	[54]
Neurogenesis	miR-124	Downregulated after stroke;shows pro-proliferative effect on SVZ stem cells	[55]
	miR-17-92 cluster	Upregulated after stroke; suppresses PTEN and promotes neural stem cell proliferation	[56]
Oligodendrogenesis	miR-9; miR-200b	Downregulated after stroke;regulate oligodendrogenesis via targeting of SRF	[57]
	miR-146a	Upregulated after stroke;enhances OPC differentiation when overexpressed	[58]

* miR denotes microRNA, BDNF brain-derived neurotrophic factor, VEGF vascular endothelial growth factor, BBB blood–brain barrier, SVZ sub-ventricular zone, PTEN phosphatase and tensin homolog, SRF serum response factor, OPC oligodendrocyte progenitor cell.
